# Alexander disease in a dog: case presentation of electrodiagnostic, magnetic resonance imaging and histopathologic findings with review of literature

**DOI:** 10.1186/s12917-015-0393-x

**Published:** 2015-05-19

**Authors:** Marcin Wrzosek, Elżbieta Giza, Marta Płonek, Przemysław Podgórski, Marc Vandevelde

**Affiliations:** Department of Internal Medicine and Clinic of Horses, Dogs and Cats, Faculty of Veterinary Medicine, Wroclaw University of Environmental and Life Sciences, pl. Grunwaldzki 47, 50-366 Wrocław, Poland; Department of General and Interventional Radiology and Neuroradiology, Wrocław Medical University, ul. Borowska 213, 50-556 Wrocław, Poland; NeuroCenter, Department of Clinical Veterinary Medicine, University of Berne, Bremgartenstrasse 109A, 3001 Berne, Switzerland

**Keywords:** Rosenthal fibers, Leucodystrophy, Electrodiagnostic, Magnetic resonance imaging, Dog

## Abstract

**Background:**

Alexander disease is a rare neurodegenerative disorder that has not often been described in dogs. None of the existing descriptions include electrodiagnostic or magnetic resonance imaging workup. This is the first presentation of the results of an electrodiagnostic evaluation including electromyography, motor nerve conduction velocity, F-wave, the brainstem auditory evoked response and magnetic resonance imaging of a dog with Alexander disease.

**Case presentation:**

A six month old male entire Bernese mountain dog was presented with central nervous system symptoms of generalized tremor, general stiffness, decreased proprioceptive positioning, a reduced menace response, decreased physiological nystagmus, myotonic spasms and increased spinal reflexes which progressed to lateral recumbency. The electromyography revealed normal muscle activity and a decreased motor nerve conduction velocity, temporal dispersion of the compound muscle action potential, prolonged F-wave minimal latency, lowered F-ratio, decreased latency, and lowered amplitude of the brainstem auditory evoked potentials. The magnetic resonance imaging examination revealed ventriculomegaly and linear hyperintensity on the border of the cortical grey and white matter. The histopathological examination confirmed the presence of diffuse degenerative changes of the white matter throughout the neuraxis. A proliferation of abnormal astrocytes was found at the border between the white matter and cortex. There was also a massive accumulation of eosinophilic Rosenthal fibers as well as diffuse proliferation of abnormally large astrocytes and unaffected neurons.

**Conclusion:**

This is the first histopathologically confirmed case of Alexander disease in a dog with a full neurological workup. The results of the electrodiagnostic and magnetic resonance imaging examinations allow for a high-probability antemortem diagnosis of this neurodegenerative disorder in dogs.

## Background

Alexander Disease (AD) is a rare neurodegenerative disorder that has been recognized in several species. Previous reports describe cases in humans [1-4], dogs [5-7], and sheep [8]. In total, 11 dogs have been diagnosed with AD, including five Bernese mountain dogs (BMD), one BMD crossbreed, one Maltese/ Shih-tzu crossbreed, two Labrador retrievers, one Scottish Terrier, one miniature poodle and one Chihuahua [5,6,8-13].

The classic histologic lesions in AD consist of a large number of the so-called Rosenthal fibers (RF), distributed around the vessels in the white matter as well as subpial and subependymal areas [2]. According to morphologic studies, the fibers consist of protein aggregates of glial fibrillary acidic protein (GFAP), αB-crystallin, heat shock protein (hsp-27) and ubiquitin in astrocytic processes throughout the central nervous system. In humans, AD can be classified as: neonatal, infantile, juvenile and adult, depending on the onset of the patient’s neurological deterioration [2].

The objective of the present article is to describe the clinical, electrodiagnostic, magnetic resonance and pathologic findings in a case of AD in a BMD.

## Case presentation

A six month old male entire BMD presented to the Wroclaw University of Environmental and Life Sciences at the Department of Internal Medicine with a Clinic of Horses, Dogs and Cats, with a history of knuckling and tremors in the hind limbs. The symptoms slowly progressed over a period of 2 months to problems with balance, incoordination, generalized ataxia, drifting to the left, head tilt to the left, knuckling on all four limbs, an aversion to touch, a reluctance to move and excessive fear. At the time of presentation, the general examination revealed an increased body temperature (40.5°C) and reddened mucous membranes. The neurological examination revealed a lateral recumbency, general stiffness, normal mentation, decreased proprioceptive positioning on all four limbs, a reduced menace response, decreased physiological nystagmus, myotonic spasms, and increased spinal reflexes. A diffuse central nervous system (CNS) lesion was suspected. There were three differential diagnoses reached: metabolic or degenerative encephalomyelopathy and an infectious disease. A complete morphological and biochemical blood examination revealed no changes.

**Figure 1:**
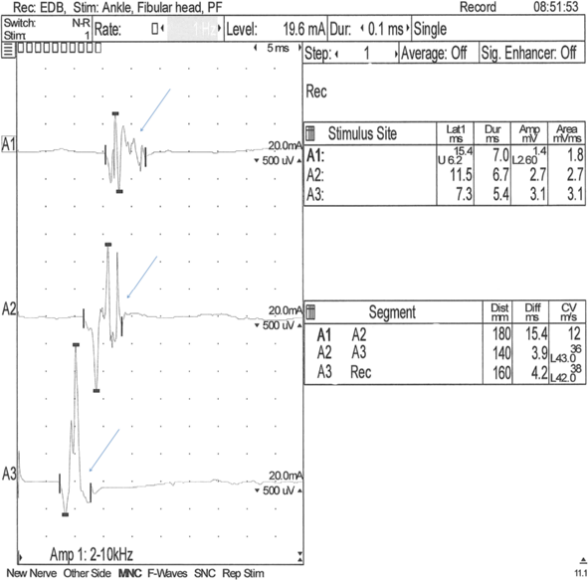
Motor nerve conduction velocity (MNCV) obtained from the left sciatic/tibial nerve. The stimulation points: proximal - A1: caudal to the greater trochanter of the femur, middle - A2: at the level of the stifle and caudal to the distal end of the femur and above the hock, distal - A3: lateral to the insertion of the gastrocnemius tendon of the tuber calcanei. The recording electrode was placed in the plantar interosseus muscle of the left foot. Decreased conduction velocity (12–38 m/sec), prolonged CMAP latency and temporal dispersion (arrows).

**Figure 2:**
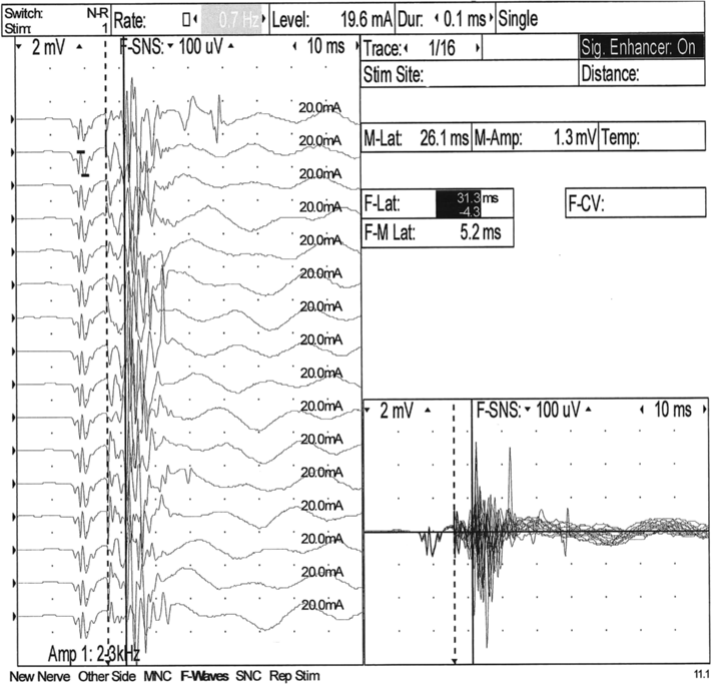
F-wave obtained from the left sciatic/tibial nerve. Prolonged F-wave latency (30,5 ms) in comparison to the expected maximum F-wave latency (21,93 ms).

Electrophysiological assessment was carried out under general anesthesia at an ambient temperature of 22°C with the use of the Nicolet Viasys Healthcare portable electrodiagnostic equipment, with 11.0 version of Viking Quest software. The body temperature dropped to 38.0 after the administration of premedication and maintained at that level during anesthesia. Electromyography (EMG) was performed by intramuscular insertions of the standard electromyographic concentric needle electrode and a subdermal monopolar ground electrode. EMG revealed normal insertional activity and normal resting activity in the tested muscles. The motor nerve conduction velocity (MNCV) and F-wave assessment were obtained from the left sciatic and tibial nerves, with the use of stimulating needle electrodes, a recording concentric needle electrode and a ground electrode, which was placed between the stimulating and recording electrodes. The MNCV was measured at three points: proximal (A1) - caudal to the greater trochanter of the femur, middle; (A2) - at the level of the stifle, caudal to distal end of the femur; (A3) distal - above the hock, lateral to the insertion of the gastrocnemius tendon of the tuber calcanei. The recording electrode was placed in the plantar interosseus muscle of the left foot. The results were compared with those of 6 month old healthy dogs [14,15] and showed a decreased conduction velocity (12–38 m/s compared with 53 ± 2.8 m/s in normal dog). The proximal latency of the compound muscle action potential (CMAP) in the BMD was 15,4 ms in comparison to 8,4 ± 0,4 ms in the normal dog [16]. The middle latency was 11,5 ms vs 6,2 ± 0,3 ms and the distal latency was 7,3 ms vs 3,4 ± 0,1 ms [16]. The duration of CMAP was also prolonged as follows: proximal latency 7 ms (reference 4,9 ± 0,4 ms), middle latency 6,7 ms (ref. 4,6 ± 0,4 ms) and distal latency 5,4 ms (ref. 4,4 ± 0,3 ms) [16]. This which is indicative of temporal dispersion (Figure 1). Sensory nerve conduction was not performed. The F-wave evaluation was based on the measurement of the minimal latency of the recorded F-waves in comparison to the expected minimum F-wave latency calculated according to the following formula: 3.45 + 0.33 × limb length [cm] for the sciatic/tibial nerve [17]. The limb length (56 cm) was measured as a distance between the greater trochanter of the femur and the distal tip of the 3rd digit. The measured minimum F-wave latency was prolonged (30.5 ms) when compared to the expected minimum F-wave latency (21,93 ms) (Figure 2). The F ratio of the sciatic/tibial nerve was calculated according to the formula: (LatF–LatM-1)/2LatM [18] and compared to the 0.883 ± 0.052 reference value at the stifle. The F ratio was 0.446, which is below the reference range described above.

**Figure 3:**
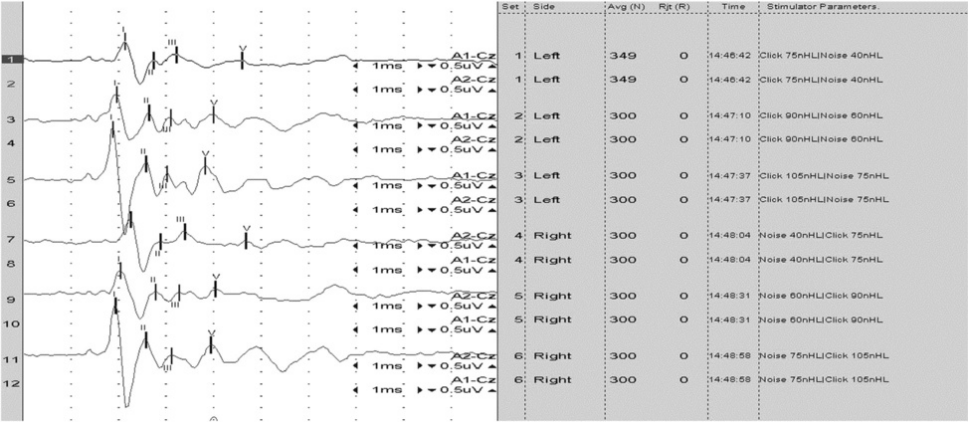
The BAER recording from the Bernese mountain dog with Alexander disease. Waves I, II, III and V were identified at 75, 90 and 105 dB for both left (recordings 1, 3 and 5) and right (recordings 7, 9, 11) ears.

Prior to the brainstem auditory evoked response (BAER) evaluation, an otoscopic examination was carried out in the dog to exclude any ear canal obstructions. The dog’s BAER was recorded (Figure 3) with the reference electrodes placed at the mastoid processes of both ears. The control group was retrospectively formed BAER recordings of our facility, from 20 healthy dogs of various breeds (8 females, 12 males), with a mean weight of 32 kg and a mean age of 6 months, using the same recording protocol as the BMD. The mean amplitude and latency of each wave was calculated and the results were compared with those obtained from the BMD. Wave latency differences between the dog and the control group were greater than 0.5 ms (shorter wave latencies in the studied dog) at 90 dB for wave III and V as well as at 105 dB for wave V in the left ear. Wave amplitudes in the BMD were generally smaller than in the control group. Wave II amplitude differences greater than 1 μV were present in both ears at all three sound intensities in the subject.

**Figure 4:**
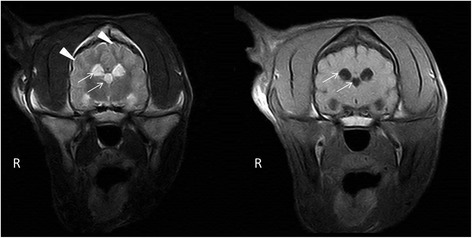
Transversal MRI T2W, T1W images. A symmetrical, bilateral ventriculomegaly of the lateral and the third ventricles (arrows), with laminar hyperintensity visible on T2W images on the border of the grey and white matter (arrowheads).

**Figure 5:**
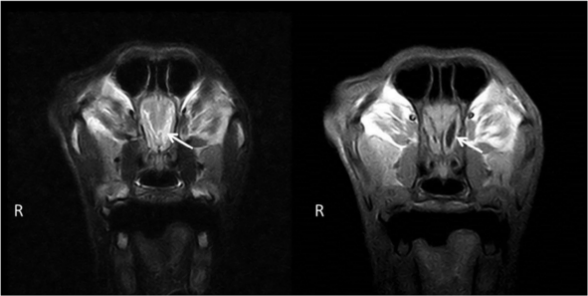
Transversal MRI T2W, T1W images. An asymmetrical, bilateral distension of the rostral recesses where the left recess is considerably larger (arrow).

**Figure 6:**
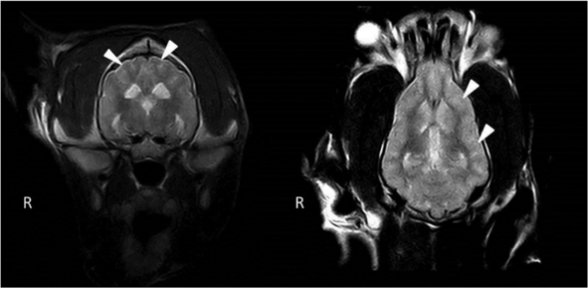
Transversal and dorsal MRI T2W images. A laminar hyperintensity is visible on T2W images on the border of the grey and white matter (arrowheads).

Magnetic resonance imaging (MRI) was performed using a low-field scanner (0,2 T system, Esaote Vet MR, Genova, Italy). Imaging parameters, sequences, and planes were tailored according to the patient size. Sequences included sagittal, transverse plane T1- and T2-weighted (T1W, T2W) images, dorsal plane T2W and transverse plane contrast enhanced T1W images (following an injection of 0,1 mmol/kg of gadobenate dimeglumine ^a^). The MRI changes included mild generalized symmetric distension of the cerebral ventricular system (ventriculomegaly) (Figure 4), and asymmetric distension of the recessus rostralis (left > right) (Figure 5). No features of increased intracranial pressure, indicative of compensatory hydrocephalus due to neuronal tissue loss (ex vacuo), were found. Linear, symmetrical, bilateral T2W hyperintense signal was observed on the border between the white and gray matter in the cerebral cortex of the frontal, temporal and occipital lobes (Figures 4 and 6). No pathological contrast enhancement was found. Three differential diagnoses were reached: leucodystrophy, other degenerative encephalopathies or metabolic disease. Cisternal cerebro-spinal fluid (CSF) analysis showed an elevated total protein concentration of 35 mg/dl (reference range <25 mg/dl) with no pleocytosis. Due to a bad prognosis, the owners decided to euthanize the dog.

**Figure 7:**
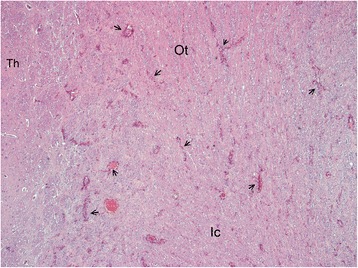
Overview histological image in the forebrain at the level of the Thalamus (Th). Diffuse pale staining and vacuolation of the white matter is seen in the internal capsule (Ic) and Optic tract (Ot). The walls of many blood vessels (arrows) in the white matter appear thickened. HE, 10X.

**Figure 8:**
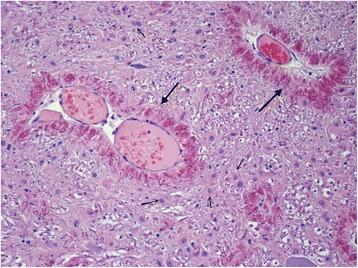
White matter of the cerebellum. Blood vessels (large arrows) are surrounded by a thick layer of short perpendicularly arranged strongly stained Rosenthal fibers. In the surrounding white matter many prominent astrocytes with large cytoplasm (small fibers) are present. HE, 200 X.

**Figure 9:**
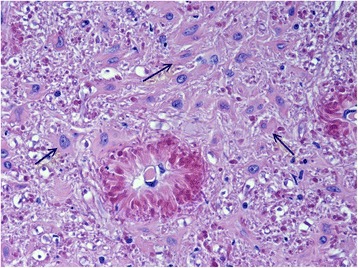
White matter in brainstem. High power magnification shows intensely eosinophilic fibrinoid like droplets in the perivascular Rosenthal fibers. There is also marked accumulation of many abnormal astrocytes (arrows) with large smooth cytoplasm. HE 400 X.

**Figure 10:**
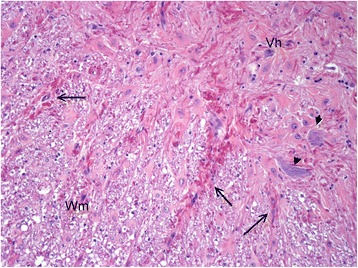
Spinal cord. In the ventral horn (Vh) massive diffuse accumulation of strongly eosinophilic Rosenthal fibers. 2 Neurons are depicted by short arrows. Intramedullary nerve roots (large arrows) extending in the white matter (Wm) also contain many Rosenthal fibers. HE, 100X.

Gross pathological examination of the cerebrum and spinal cord was unremarkable. Sections (5 μm) were stained with haematoxylin and eosin (HE), Luxor fast blue (LFB) and toluidine blue, using the periodic acid-Schiff (PAS) and Bielschowsky silver impregnation methods. The histopathological examination of the brain and spinal cord revealed diffuse degenerative changes of the white matter throughout the neuraxis, with a varying degree of severity (Figure 7). A proliferation of astrocytes with an open faced nucleus and large glassy eosinophilic to pale cytoplasm was observed in the affected areas (Figure 8). In the forebrain, the proliferation of the abnormal astrocytes tended to be more intense at the border between the white matter and the cortex. A massive accumulation of eosinophilic RF was noted presumably in the astrocytic endfeet especially around blood vessels, (Figure 8). These fibrous accumulations often contained intensely stained eosinophilic droplets reminiscent of fibrinoid material (Figure 9). Involvement of brainstem and spinal cord gray matter was also noted with marked diffuse accumulation of RF and diffuse proliferation of abnormally large astrocytes, unaffecting neurons (Figure 10). The Rosenthal fibers often extended along the ventral horn axons traversing the spinal cord white matter (Figure 10). The fibers were also found in subpial and some subventricular locations.

## Discussion

AD was first described in humans by Stewart Alexander in 1949 [19]. In humans, four clinical forms of the disease have been described: neonatal, infantile, juvenile and adult, depending on the onset of the patient’s neurological deterioration [2]. In 2001, a study by Brenner et al. linked the disease to dominant heterozygous point mutations predicting non-conservative amino acid changes on the GFAP gene. This was the first example of a primary genetic astrocyte disorder. The presence of RF (which are protein aggregates of glial fibrillary acidic protein, αB-crystallin, heat shock protein (hsp-27) and ubiquitin in astrocytic processes throughout the central nervous system) is characteristic of the disease [3]. Although Rosenthal fibers may be present in other disorders (tumours, glial scars, multiple sclerosis), their distribution in Alexander disease is unique because they occur not only around blood vessels in the white matter, but also in subpial and subependymal areas [2].

In veterinary medicine, there have been few reports of Alexander disease. To date, the disease has been described in dogs and sheep. Reports in sheep, suggest a hereditary etiology [8,20]. Although it was impossible to follow the pedigree of the presented dog, an inherited mode of transmission should be considered based on previous reports of the disease in this breed [5,6,12]. An autosomal dominant mode of inheritance is suspected in humans and other affected dog breeds [6,10,11,13].

It is postulated that a metabolic defect of astrocytes plays a primary role in the pathogenesis of AD. An overexpression of abnormal GFAP appears as a result of a mutation, and leads to filament disorganization, decreased solubility, possibly ineffective degradation and clearance of the aberrant protein [8]. Stress may be an initiating factor for the precipitation of the mutant GFAP protein and the production of ubiquitin and heat shock proteins (αB-crystallin, hsp-27) accumulating as RF. The astrocytic dysfunction affects oligodendrocyte survival, leading to abnormal myelin formation, maintenance [21], and thus to the clinical disseminated appearance of the disease.

Clinical signs reported in dogs with AD were similar to the ones described here and included ataxia, head tremor, posterior weakness, trembling of hind limbs, deteriorating to tetraparesis or –plegia within 3–4 weeks, lateral recumbency and normal to exaggerated spinal reflexes. Less commonly described symptoms include head tilt with normal mentation, circling, nystagmus, pupillotonia and deterioration of consciousness leading to coma [6,7,12]. Our case presented a condition and progression of signs similar to the previously described BMD [5,6,12]. The mean age at which the onset of the clinical symptoms occurred in the all the reported BMD was 12.75 months (median 13) [5-7,12]. In other dog breeds (Scottish terrier, miniature poodle, Labrador retriever) the onset was noticed later (mean 21 months, median 24) [7,9,10], except for one Chihuahua (approx. 8 m) [6]. In this BMD, the onset of the clinical symptoms was observed earlier than in other breeds (6 months). However, in all the described cases in dogs, the age of the onset and disease progression seem to correspond to the juvenile form of AD in humans. An AD onset in mature dogs has not been reported.

To date, there has been no MRI description of AD in veterinary medicine. In 2001, van der Knaap et al. determined five MR imaging criteria of Alexander disease in humans that allow a presumptive diagnosis to be made without further histopathological confirmation. These include: extensive cerebral white matter changes with frontal predominance, a periventricular rim with high signal on T1-weighted images and low signal on T2-weighted images, abnormalities of the basal nuclei and thalami, brainstem abnormalities, and contrast enhancement of particular gray and white matter structures. Four of the five criteria have to be met for an MR imaging-based AD diagnosis. Such criteria have not been applied in veterinary medicine, and all the described reports confirmed the disease in animals histopathologically. The BMD was scanned in a low-field MRI scanner, which could be a reason for the limited imaging changes. The observed symmetrical bilateral ventriculomegaly, without features of increased ICP, was highly suggestive of a nervous tissue degeneration, pointing to a diffuse degenerative CNS disease [5]. Compensatory hydrocephalus due to various CNS degenerative and metabolic diseases has been described in veterinary literature [22-24]. A linear hyperintensity has been reported as a consequence of laminar cerebrocortical necrosis due to thiamine deficiency in calves and dogs [25,26]. It can also be found in ischemia-induced brain damage due to an increase in lactate levels and neuronal excitotoxicity. The dog underwent an MRI examination at a young age, which could be a reason for the lack of MRI abnormalities similar to those observed in humans. The value of MRI for the diagnosis of AD in veterinary medicine has yet to be determined. Although a definitive diagnosis based on extracerebral tissue biopsy is not possible, an antemortem diagnosis can be based on peripheral nerve tissue or brain biopsy [27].

Histopathological changes in Alexander disease are mainly concerned with the central nervous system white matter in both human and veterinary patients. The peripheral nerve and muscle examinations were reported in only a few human cases of this disease, showing occasional segmental demyelination, an abundance of π granules of Reich and polymorphic lipid inclusions in Schwann cells, which appeared to be myelin degradation products [28]. According to Escourolle et al., the mechanism behind peripheral nerve involvement remains unclear. Similarly, reports describing PNS involvement in veterinary patients are very limited. McGrath [9] stated that there were no abnormalities in peripheral and cranial nerves in two Labrador retrievers with AD. Also, no pathological changes were found in the nerves or skeletal muscles of a 4-year old White Alpine sheep [20]. The study conducted by Cox et al. [7] revealed abnormalities in peroneal and ulnar nerves. These included occasional degenerating fibers, characterized by myelin ovoids and balls and paranodal demyelination in 14% of nerve fibers, which was consistent with demyelinating polyneuropathy. A skeletal muscle examination revealed mild focal fiber necrosis, focal and multifocal fatty infiltration and occasional whorled fibers. Moreover, single or multiple subsarcolemmal, basophilic masses in many fibers of cranial tibial muscle were found. The correlation between the above mentioned changes and a diffuse formation of RF in the CNS remains unknown [7].

Although the pathologic examination of the peripheral nerves was not performed in the present case, the electrodiagnostic examination was indicative of demyelination that involves all the levels of the sciatic/tibial nerve, with a conduction abnormality distributed more distally along the nerve. In human patients, Nagao et al. [27] reported a delay in MNCV, suggesting peripheral nerve involvement in certain cases of fibrinoid leucodystrophy. Recently, Pareyson et al. [29] described mild neurogenic electromyographic abnormalities in 6 out of 8 cases of adult-onset Alexander disease (AOAD). In 2013, Melchionda et al. reported similar abnormalities on EMG together with MNCV findings consistent with mild motor axonal neuropathy in one patient with AOAD. Pareyson et al. found changes in the BAER examination in seven subjects with AD, involving all the waves or limited to waves I and III [29]. All of these patients had marked bulbar changes on MRI. BAER abnormalities have also been described in a six year old boy with AD resembling the juvenile form, who had prolonged latencies between waves III and V at 90 dB [30]. On the other hand, Nair [31] reported that in a 3-year-old boy with AD, auditory evoked potentials were normal, despite the presence of signal changes in the brainstem, visible on MRI. A normal auditory evoked response was recorded in a 29 year old female patient, who had no changes visible on MRI [32]. The usefulness of BAER studies has been reported in patients with other leukodystrophies (Pelizaeus-Merzbacher leukodystrophy, adrenoleukodystrophy and metachromatic leukodystrophy). The majority of those had waves III-VII absent [33]. BAER changes in the present case report differ from those described in human literature. The BMD had shorter latencies of waves III and V at 90 and 105 dB than the control group, a finding not reported in human patients. The BMD differed from the control group in terms of age (which should not have affected the results since wave latencies stabilize by the 40^th^ postnatal day as shown by Strain et al. [34]), gender (which has also been found not to affect wave latencies in dogs [35]) and head size. Although the effect of head size on the BAER is disputable in both dogs and humans, it has been found that large breed dogs have longer wave latencies than small dogs [35]. The size of the cranium was not recorded, but all dogs in the control group were of a similar body mass and age to the BMD. However, the wave latency differences may have been due to differences in the head size of the control dogs and the BMD. On the other hand Ochs et al. emphasized the usefulness of BAER in conjunction with other electrodiagnostic tests in differentiating between leukodystrophy and progressive gray matter disease in human patients. Although this test does not suffice to diagnose AD, it may be helpful in determining its severity. To the authors’ knowledge, this is the first report of the electrodiagnostic changes in a dog with Alexander disease.

## Conclusion

The presented results show the first histopathologically confirmed case of Alexander disease in a dog with a full neurological workup. Taking into account the history, breed predisposition, neurological, electrodiagnostic and magnetic resonance imaging findings, it may be possible to make an antemortem diagnosis of this neurodegenerative disorder in dogs.

## Endnote

^a^gadobenate dimeglumine - Gadovist 1.0 mmol, 604.72 mg/ml, gadobutrolum, Bayer Pharma AG, D-13342, Berlin, Germany.

## Abbreviations

AD: Alexander Disease
BMD: Bernese mountain dogs
RF: Rosenthal fibers
CMAP: Compound muscle action potential
CNS: Central nervous system
EMG: Electromyography
MNCV: Motor nerve conduction velocity
BAER: Brainstem auditory evoked response
MRI: Magnetic resonance imaging
T1W: T2W, T1- and T2-weighted
CSF: Cerebro-spinal fluid
HE: Haematoxylin and eosin
LFB: Luxor fast blue
PAS: Periodic acid-Schiff
GFAP: Glial fibrillary acidic protein
hsp-27: Heat shock protein 27
ICP: Intracranial pressure
CP: Conscious proprioception
PNS: Peripheral nervous system
